# Supplementary material for: Selectivity of Oxygen Evolution Reaction on Carbon Cloth-Supported δ-MnO2 Nanosheets in Electrolysis of Real Seawater
Source: Molecules. 2023 Jan 14;28(2):854. doi: 10.3390/molecules28020854 (PMC9863582; doi:10.3390/molecules28020854)
Supplement: Supplementary file 1 [file molecules-28-00854-s001.zip › molecules-2124456-supplementary.pdf]

Selectivity of oxygen evolution reaction on carbon cloth - supported δ-MnO_2 nanosheets in electrolysis of real seawater

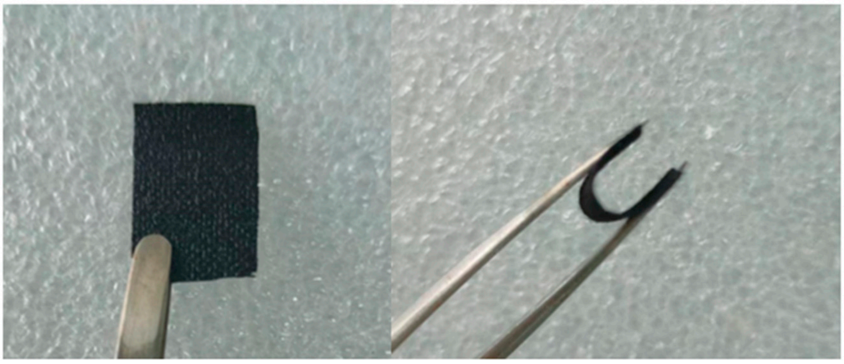

Figure S1. MnO_2/CC

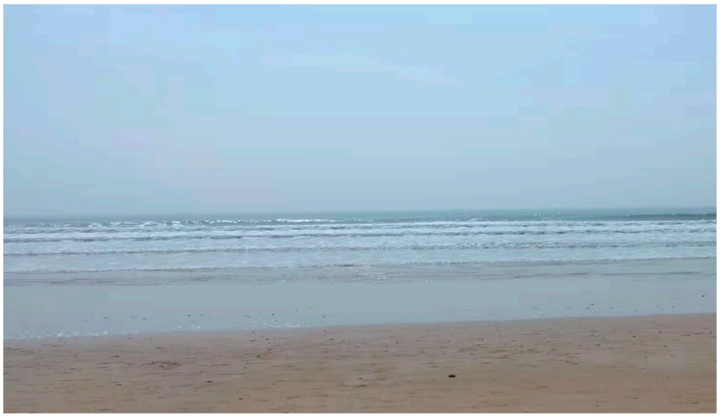

Figure S2. Real seawater collection site

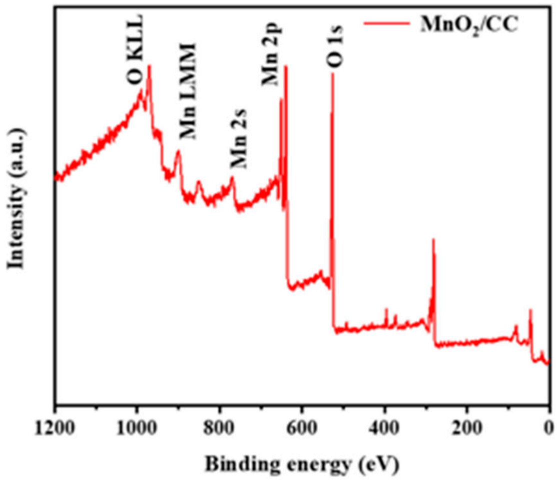

Figure S3. XPS full spectra of MnO_2/CC

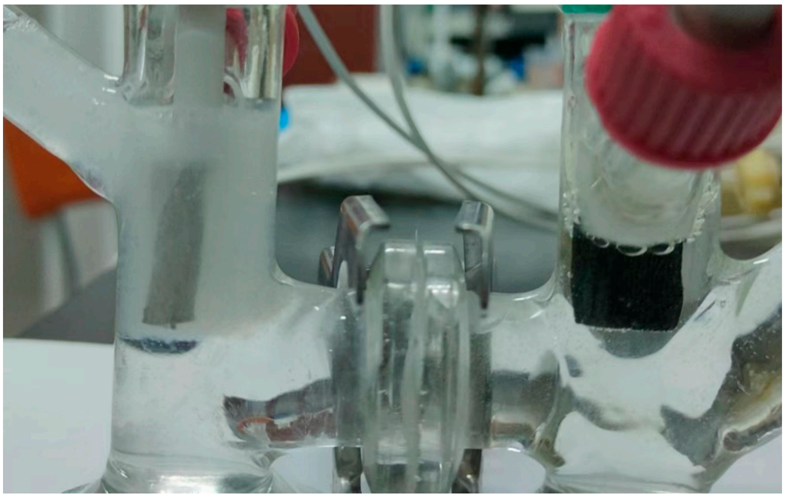

Figure S4. White precipitates are produced at the cathode during electrolysis of seawater

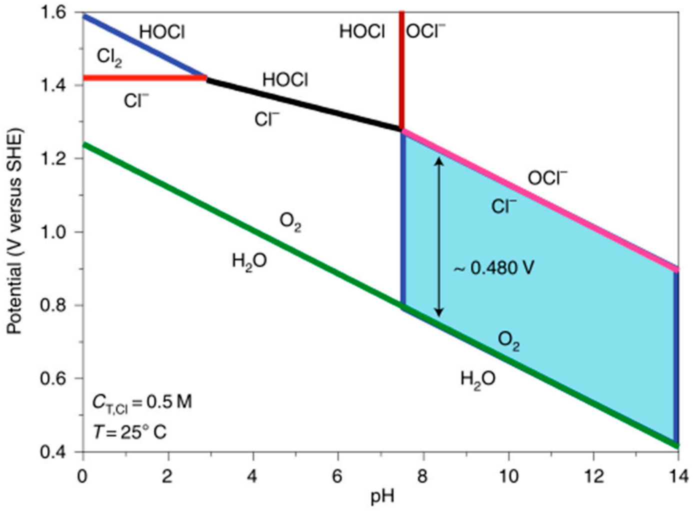

Figure S5. The Pourbaix diagram of an aqueous saline electrolyte [36].

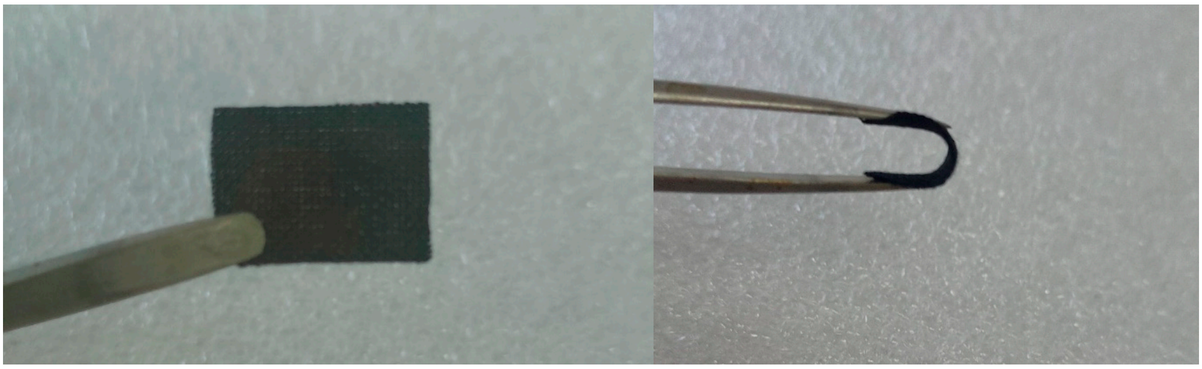

Figure S6. Constant current electrolysis experiment at the end of MnO_2/CC

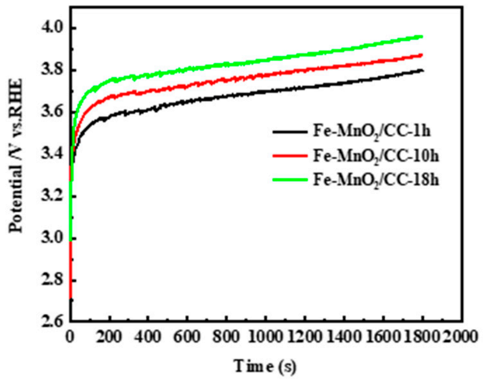

Figure S7. V-t curve of Fe-MnO2/CC sample with intercalation time of 1h, 10h and 18h at constant current of 100 mA.

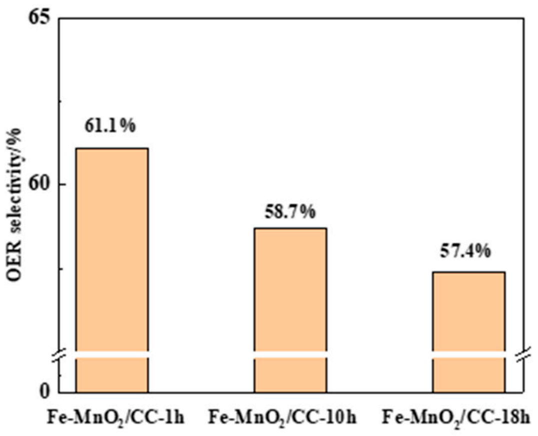

Figure S8. Fe-MnO2/CC oxygen evolution selectivity histogram at different intercalation times

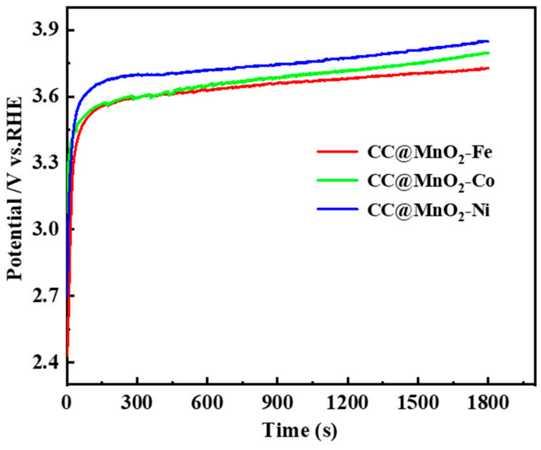

Figure S9. V-t curves of Fe-MnO2/CC, Co-MnO2/CC, Ni-MnO2/CC samples at constant current of 100 mA.

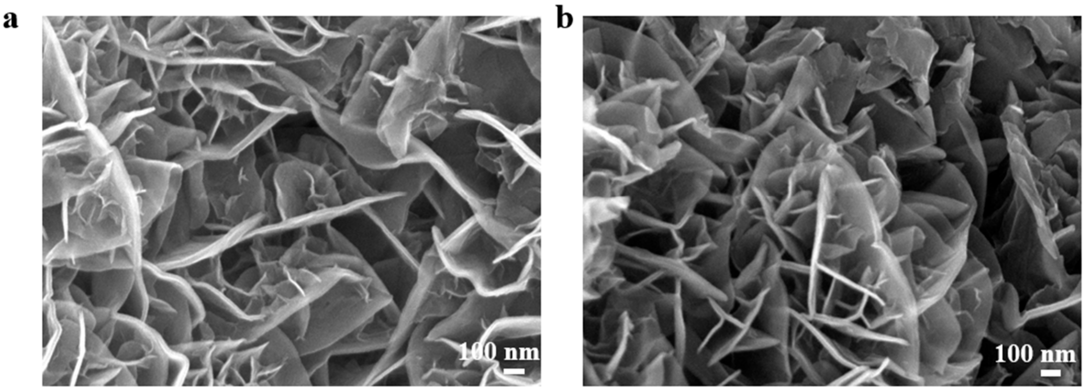

Figure S10. (a) SEM image of MnO_2/CC after electrochemical test; (b) SEM image of Fe-MnO_2/CC after electrochemical test.

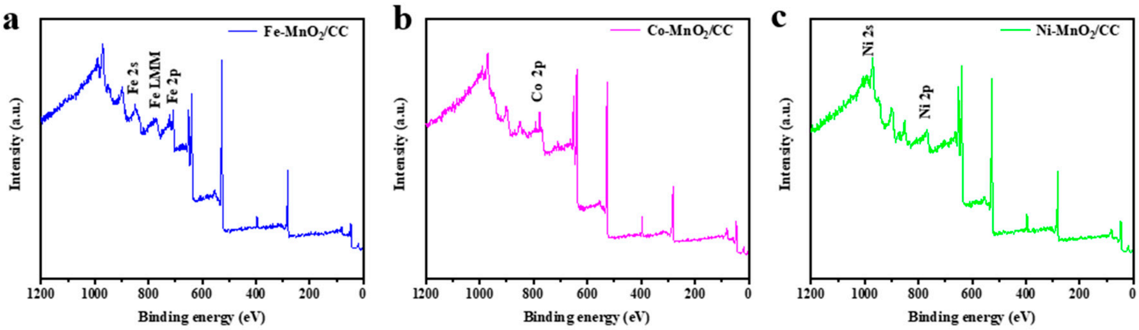

Figure S11. (a) XPS full spectra of Fe-MnO_2/CC; (b) XPS full spectra of Co-MnO_2/CC; (c) XPS full spectra of Ni-MnO_2/CC.

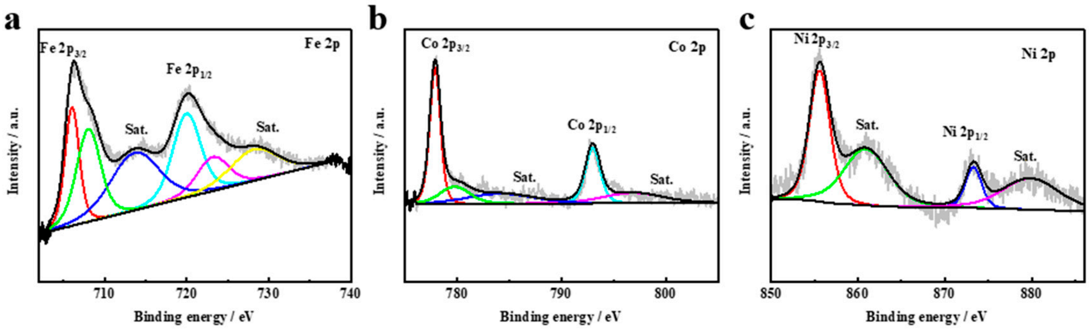

Figure S12. (a)High resolution Fe 2p XPS spectra of Fe-MnO_2/CC; (b) High resolution Co 2p XPS spectra of Co-MnO_2/CC; (c)High resolution Ni 2p XPS spectra of Ni-MnO_2/CC.

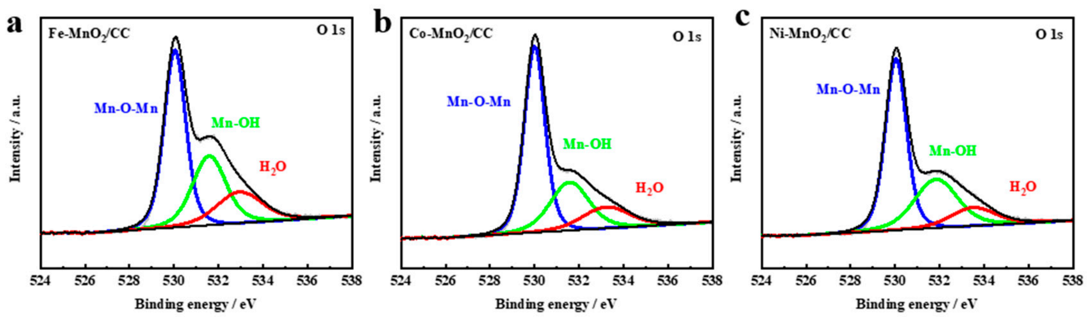

Figure S13. (a)High resolution O 1S XPS spectra of Fe-MnO_2/CC; (b) High resolution O 1S XPS spectra of Co-MnO_2/CC; (c)High resolution O 1S XPS spectra of Ni-MnO_2/CC.

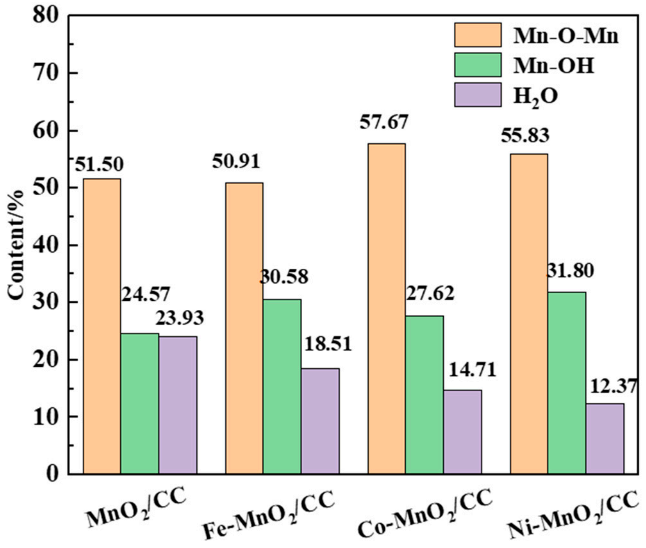

Figure S14. The contents of Mn-O-Mn, Mn-OH and H_2O in MnO_2/CC, Fe-MnO_2/CC, Co-MnO_2/CC and Ni-MnO_2/CC

Table S1. Binding energy corresponding to Mn(Ⅲ), Mn(Ⅳ) peak positions in MnO2/CC Mn2P high-resolution XPS spectra.

	Binding energy(eV)	
species	Mn $2p_{3/2}$	Mn $2p_{1/2}$
+3	641.86	653.57
+4	643.14	654.82

Table S2. Binding energy corresponding to Mn(III), Mn(IV) peak positions in Fe-MnO2/CC，Co-MnO2/CC and Ni-MnO2/CC Mn2P high-resolution XPS spectra.

Sample	species	Binding energy(eV)	
		Mn $2p_{3/2}$	Mn $2p_{1/2}$
Fe-MnO_2/CC	+3	642.19	653.68
	+4	643.16	655.06
Co-MnO_2/CC	+3	642.08	653.84
	+4	643.38	654.91
Ni-MnO_2/CC	+3	642.15	653.74
	+4	643.70	655.04

Table S3. Titration results of solution in tail gas absorption zone after constant current electrolytic seawater test. (Fe-MnO_2/CC-1h)

	1	2	3
Electrolyte volume/mL	10		
KI volume /mL	15		
Initial reading /mL	37.70	32.70	34.70
End reading /mL	45.50	40.70	42.30
Average of Na_2SO_3 consumed/mL	7.80		
nCl_2/mol	0.000156		
Total battery /C	180		
Cl^- Oxidation power /C	30.1		

Table S4. Titration results of anodic solution after constant current electrolytic seawater test. (Fe-MnO_2/CC-1h)

	1	2	3
NaOH volume/mL	10		
KI volume /mL	15		
Initial reading /mL	23.80	18.90	21.00
End reading /mL	37.70	32.70	34.70
Average of Na_2SO_3Consume/mL	13.80		
nCl_2/mol	0.000276		
Total battery /C	180		
Cl^- Oxidation power /C	39.9		
Total Cl^- oxidation power/C	70.00		
Cl^- Oxidation ratio /%	38.89		
OER selectivity/%	61.11		

Table S5. Titration results of solution in tail gas absorption zone after constant current electrolytic seawater test. (Fe-MnO_2/CC-10h)

	1	2	3
Electrolyte volume/mL	10		
KI volume /mL	15		
Initial reading /mL	34.00	31.10	33.70
End reading /mL	42.00	39.20	41.60
Average of Na_2SO_3 consumed/mL	8.00		
nCl_2/mol	0.00016		
Total battery /C	180		
Cl^- Oxidation power /C	30.9		

Table S6. Titration results of anodic solution after constant current electrolytic seawater test. (Fe-MnO_2/CC-10 h)

	1	2	3
NaOH volume/mL	10		
KI volume /mL	15		
Initial reading /mL	18.90	16.20	18.70
End reading /mL	34.0	31.10	33.70
Average of Na_2SO_3Consume/mL	15.00		
nCl_2/mol	0.003		
Total battery /C	180		
Cl^- Oxidation power /C	43.4		
Total Cl^- oxidation power/C	74.3		
Cl^- Oxidation ratio /%	41.28		
OER selectivity/%	58.72		

Table S7. Titration results of solution in tail gas absorption zone after constant current electrolytic seawater test. (Fe-MnO_2/CC-18h)

	1	2	3
Electrolyte volume/mL	10		
KI volume /mL	15		
Initial reading /mL	39.80	36.40	35.50
End reading /mL	46.80	43.30	42.30
Average of Na_2SO_3 consumed/mL	6.90		
nCl_2/mol	0.000138		
Total battery /C	180		
Cl^- Oxidation power /C	26.6		

Table S8. Titration results of anodic solution after constant current electrolytic seawater test. (Fe-MnO_2/CC -18h)

	1	2	3
NaOH volume/mL	10		
KI volume /mL	15		
Initial reading /mL	22.50	19.00	18.30
End reading /mL	39.80	36.40	35.50
Average of Na_2SO_3Consume/mL	17.30		
nCl_2/mol	0.00346		
Total battery /C	180		
Cl^- Oxidation power /C	50.0		
Total Cl^- oxidation power/C	76.6		
Cl^- Oxidation ratio /%	42.56		
OER selectivity/%	57.44		

Table S9. Titration results of solution in tail gas absorption zone after constant current electrolytic seawater test. (MnO_2/CC)

	1	2	3
Electrolyte volume/mL	10		
KI volume /mL	15		
Initial reading /mL	36.90	35.20	36.10
End reading /mL	43.50	41.70	42.50
Average of Na_2SO_3 consumed/mL	6.50		
nCl_2/mol	0.00013		
Total battery /C	180		
Cl^- Oxidation power /C	25.0		

Table S10. Titration results of anodic solution after constant current electrolytic seawater test. (MnO_2/CC)

	1	2	3
NaOH volume/mL	10		
KI volume /mL	15		
Initial reading /mL	24.80	22.70	23.80
End reading /mL	36.90	35.20	36.10
Average of Na_2SO_3Consume/mL	12.30		
nCl_2/mol	0.000276		
Total battery /C	180		
Cl^- Oxidation power /C	35.6		
Total Cl^- oxidation power/C	60.6		
Cl^- Oxidation ratio /%	33.67		
OER selectivity/%	66.333		

Table S11. Titration results of solution in tail gas absorption zone after constant current electrolytic seawater test. (Co-MnO_2/CC-1h)

	1	2	3
Electrolyte volume/mL	10		
KI volume /mL	15		
Initial reading /mL	34.50	35.40	34.80
End reading /mL	42.40	43.70	42.90
Average of Na_2SO_3 consumed/mL	8.10		
nCl_2/mol	0.000162		
Total battery /C	180		
Cl^- Oxidation power /C	31.4		

Table S12. Titration results of anodic solution after constant current electrolytic seawater test. (Co-MnO_2/CC-1h)

	1	2	3
NaOH volume/mL	10		
KI volume /mL	15		
Initial reading /mL	20.10	21.20	20.50
End reading /mL	34.50	35.40	34.80
Average of Na_2SO_3Consume/mL	14.30		
nCl_2/mol	0.000286		
Total battery /C	180		
Cl^- Oxidation power /C	42.2		
Total Cl^- oxidation power/C	73.6		
Cl^- Oxidation ratio /%	40.90		
OER selectivity/%	59.10		

Table S13. Titration results of solution in tail gas absorption zone after constant current electrolytic seawater test. (Ni-MnO_2/CC-1h)

	1	2	3
Electrolyte volume/mL	10		
KI volume /mL	15		
Initial reading /mL	33.30	34.40	39.70
End reading /mL	39.50	40.80	46.30
Average of Na_2SO_3 consumed/mL	6.40		
nCl_2/mol	0.000128		
Total battery /C	180		
Cl^- Oxidation power /C	24.7		

Table S14. Titration results of anodic solution after constant current electrolytic seawater test. (Ni-MnO_2/CC -1h)

	1	2	3
NaOH volume/mL	10		
KI volume /mL	15		
Initial reading /mL	19.50	20.60	25.90
End reading /mL	33.30	34.40	39.70
Average of Na_2SO_3Consume/mL	13.80		
nCl_2/mol	0.000276		
Total battery /C	180		
Cl^- Oxidation power /C	40.1		
Total Cl^- oxidation power/C	64.8		
Cl^- Oxidation ratio /%	36.00		
OER selectivity/%	64.00		
